# Application of Adaptive Filtering Based on Variational Mode Decomposition for High-Temperature Electromagnetic Acoustic Transducer Denoising

**DOI:** 10.3390/s22187042

**Published:** 2022-09-17

**Authors:** Shuaijie Zhao, Jinjie Zhou, Yao Liu, Jitang Zhang, Jie Cui

**Affiliations:** School of Mechanical Engineering, North University of China, Taiyuan 030051, China

**Keywords:** EMAT, high temperature, SNR, thickness measurement

## Abstract

In high-temperature environments, the signal-to-noise ratio (SNR) of the signal measured by electromagnetic acoustic transducers (EMAT) is low, and the signal characteristics are difficult to extract, which greatly affects their application in practical industry. Aiming at this problem, this paper proposes the least mean square adaptive filtering interpolation denoising method based on variational modal decomposition (AFIV). Firstly, the high-temperature EMAT signal was decomposed by variational modal decomposition (VMD). Then the high-frequency and low-frequency noises in the signal were filtered according to the excitation center frequency. Following the wavelet threshold denoising (WTD) for the noise component after VMD decomposition was carried out. Afterward, the noise component and signal component were connected by an adaptive filtering process to achieve further noise reduction. Finally, cubic spline interpolation was used to smooth the noise reduction curve and obtain the time information. To verify the effectiveness of the proposed method, it was applied to two kinds of ultrasonic signals from 25 to 700 °C. Compared with VMD, WTD, and empirical mode decomposition denoising, the SNR was increased by 2 times. The results show that this method can better extract the effective information of echo signals and realize the online thickness measurement at high temperature.

## 1. Introduction

At present, boilers, reactors, and high-temperature pipelines are widely used in industry. Due to the influence of working conditions, they often work under high-temperature environments. In addition, due to corrosion caused by chemical action, the thickness of the equipment will gradually decrease, and the strength of the equipment will be weakened, which will ultimately affect the safety of the equipment. Compared with traditional piezoelectric ultrasound technology, an electromagnetic acoustic transducer (EMAT) is suitable for non-destructive testing in high temperature, online, mobile, and other harsh environments because it has the characteristics of non-contact, no coupling agent, and low requirements on the roughness of metal surfaces [1,2]. Therefore, the application of EMAT non-destructive testing technology to the online real-time measurement of high-temperature equipment wall thickness and internal damage detection has important engineering application value. This technology can not only detect corrosion defects as early as possible, but also save on inspection cost and improve inspection efficiency [3]. However, its energy conversion efficiency is low, the amplitude of echo signal is small, and many spikes and mutations often cause the useful signal to be drowned by noise. At the same time, for high-temperature equipment, the increase in ultrasonic propagation attenuation coefficient and the increase in high-temperature structure noise lead to the decrease in the signal-to-noise ratio (SNR) of the detection echo, so it is difficult for EMAT to achieve effective thickness measurements in high-temperature environments. To improve the SNR of EMAT systems, the denoising signal processing method is the key step to improving the effectiveness and reliability of the system. However, for the high-temperature EMAT signal, the signal SNR after processing by the existing noise reduction methods is relatively low, and the echo signal is buried in the noise signal. As the temperature rises, the noise becomes stronger. The extraction process of echo information is only based on the data point itself, without considering the influence of the data change trend. Therefore, a new denoising method is needed to solve these problems.

To solve the problem of low SNR in ultrasonic detection, scholars have conducted a lot of research. Fourier transform is widely used as the simplest signal processing method. However, this method does not provide time domain information. The wavelet threshold denoising (WTD) method was proposed by Donoho et al. [4] in 1995. It makes up for the insufficient time–frequency resolution of short-time Fourier transform (STFT) and has been widely used in the field of noise reduction. Legendre et al. [5] proposed a wavelet-based method for analyzing received ultrasonic signals during the detection of reinforced composites. At present, many scholars directly use the WTD method to denoise the decomposed components of the signal, respectively, and then reconstruct the processed denoising signal. Although this method is simple and easy to operate, it does not take into account the characteristics of spectrum distribution of each component after signal decomposition. Some components may be partially or completely Gaussian white noise, which does not effectively remove the noise component and retain the useful component. Huang et al. [6] proposed an empirical mode decomposition (EMD) method, which decomposes the signal time–domain waveform into multiple intrinsic mode functions (IMF) and a cumulative form of residual quantity according to different frequency–domain sub-bands. Sun et al. [7] used EMD to process ultrasonic signals, suppress noise and enhance defect signals and carried out experimental verification on stainless steel tubes with defects. Xu et al. [8] proposed an ultrasonic-guided wave signal processing method combining the EMD method and STFT for the non-destructive evaluation of layered composites. However, EMD decomposition has disadvantages such as modal aliasing and end-point effects due to its empirical nature and no strict mathematical derivation. Wu and Huang [9] proposed ensemble empirical mode decomposition (EEMD), which can suppress mode aliasing. However, Gaussian white noise is introduced into the EEMD algorithm and a pseudo-component is generated in the decomposition result. Zhang et al. [10] proposed an extraction method of laser ultrasonic defect signals based on step-by-step EEMD and joint solution. Laser ultrasonic detection was carried out on the defective of 45# steel sample, the defect signals were extracted from the collected detection signals, and the error was within 3%. However, EEMD does not essentially solve the disadvantages of modal aliasing and end-point effects. Dragomiretskiy et al. [11] proposed the method of variational modal decomposition (VMD) in 2014, that is, in the process of obtaining decomposition components, the center frequency and bandwidth of each component are determined by iteratively searching the optimal solution of the variational model. Thus, the frequency domain subdivision of signals and the effective separation of each component can be realized adaptively. This method has strong adaptability and good robustness to strong noise, which is especially suitable for processing non-stationary signals. Because an ultrasonic signal is a kind of multimode aliasing signal, different modes have different ultrasonic characteristics, so the VMD method can be used to decompose the body wave. Si et al. [12] proposed an improved VMD fusion wavelet method to denoise the signal of EMAT, which can suppress high-frequency narrowband noise and is suitable for EMAT signals under high lift-off detection conditions. Abdessalem et al. [13] proposed an improved VMD algorithm to improve the resolution of ultrasonic signals and facilitate the detection and location of defect echoes. So far, the VMD algorithm has been widely applied in various fields [14,15,16,17]. In terms of high-temperature EMAT, Kogia et al. [18] realized SH0-guided wave detection of stainless steel and carbon steel at 600 °C by optimizing the water-cooling scheme of the high-temperature EMAT. Shi et al. [19] used chirp pulse compression technology to conduct EMAT tests on forgings at different temperatures from 92 to 730 °C. This method can greatly improve the lift-off, SNR, range resolution, and inspection efficiency. In terms of noise barriers, Basili et al. [20] proposed a strategy employing a hysteretic vibration absorber to mitigate the noise barrier vibrations due to train passage in high-speed lines.

Modern filtering and noise reduction methods, such as Kalman filtering, Wiener filtering, particle filtering [21], and adaptive filtering, are also common noise reduction methods. The adaptive filter does not completely depend on the prior statistical characteristics of the input signal when designing the filter and does not have the problem of threshold selection in some denoising methods. The core of the adaptive filter is the adaptive algorithm, which adaptively adjusts parameters and updates the result of signal estimation to optimize the output.

As can be seen from the above references, the existing classical noise reduction algorithms based on modal decomposition class, and some improved algorithms, often only carry out noise reduction independently within different components, while ignoring the relationship between the noise among components [22,23]. Based on the above shortcomings, this paper proposes the least mean square adaptive filtering interpolation denoising method based on VMD (AFIV).

Firstly, the method and principle of this paper are introduced. On this basis, an experimental system is designed to acquire electromagnetic ultrasonic signals at different temperatures. Finally, in order to verify the feasibility and effectiveness of the proposed method, the noise reduction effects of VMD, WTD, EMD, and AFIV on EMAT signals at different temperatures are compared and discussed.

## 2. Basis Theory

### 2.1. The Principle of EMAT

**Figure 1:**
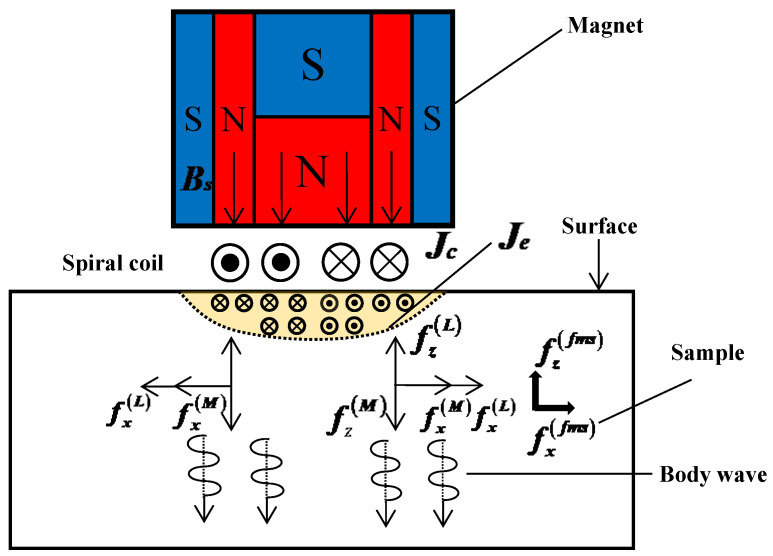
Basic principles and components of EMAT.

Figure 1 shows the generation principle of the electromagnetic ultrasonic wave. Two key components of the EMAT are metal coils and permanent magnets. The permanent magnet generates a vertical static magnetic field *B*_s_ around the metal coil. When the high-frequency alternating current with the density of *J*_c_ flows in the coil, an alternating magnetic field generates, and an eddy current *J*_e_ shows up in the surface of the tested sample. For the non-ferromagnetic materials, the eddy currents generate Lorentz forces $f^{\left(L\right)}$ under the action of a bias magnetic field provided by a permanent magnet and an alternating magnetic field. The Lorentz force causes localized vibrations within the test sample, which in turn generate ultrasonic waves. When the measured material is ferromagnetic materials, an ultrasonic wave is generated under the joint action of Lorentz force $f^{\left(L\right)}$, magnetostrictive force $f^{\left(fms\right)}$, and magnetization force $f^{\left(M\right)}$ [24,25,26,27]. All are beneficial to improve the excitation efficiency of ultrasound. The acoustic reception is based on the reverse process.

In the received high-temperature EMAT signal, there are electronic noises, structure noises, and intense noises generated by huge radiant heat. All these noises make the echo signal containing useful information buried in the noise, thus unable to provide detection capability. In order to solve the above problems, it is urgent to find an effective noise reduction method. Therefore, the following noise reduction theory is deeply studied in this paper.

### 2.2. Variational Mode Decomposition (VMD)

VMD is often used to compress noise, which works as follows:

(1) Create a variational problem. In the VMD algorithm, the original signal f is decomposed into K IMF $u_{k}\left(t\right)$, whose center frequency is $\omega_{k}\left(t\right)$. The expression is $u_{k}\left(t\right)=A_{k}\left(t\right)\cos\left(\phi_{k}\left(t\right)\right)$. Where, $A_{k}\left(t\right)$ is the instantaneous amplitude of $u_{k}\left(t\right)$, $\phi_{k}\left(t\right)$ is the phase, and $u_{k}\left(t\right)$ is considered to be a harmonic signal with an amplitude of $A_{k}\left(t\right)$ and a frequency of $\omega_{k}\left(t\right)$. To minimize the sum of the estimated bandwidths of each modal function, set up a constrained variational problem as shown below [11]:

(1) $$\left\{{}_{\sum_{k=1}^{K}u_{k}=f}^{\underset{\left\{u_{k}\right\},\left\{\omega_{k}\right\}}{\min}\left(\sum_{k=1}^{K}{\left\|\partial_{t}\left[\left(\delta \left(t\right)+\frac{j}{\pi t}\right)\times u_{k}\left(t\right)\right]e^{-j\omega_{k}t}\right\|}_{2}^{2}\right)}\right.$$

where K is the number of modes, and $\omega_{k}\left(t\right)$ is the center frequency of $u_{k}\left(t\right)$. $\delta \left(t\right)$ is the unit impulse function, $\partial_{t}$ is the partial derivative of t with respect to the unit impulse function, *j* is the imaginary number, and ${\left\|\right\|}_{2}$ is the L2 norm.

(2) Solve the variational problem. The augmented Lagrange function L is introduced to transform the constrained problem into an unconstrained problem:

(2) $$\begin{array}{l} L\left(\left\{u_{k}\right\},\left\{\omega_{k}\right\},\lambda \right)=\alpha \sum_{k=1}^{K}{\left\|\partial_{t}\left[\left(\delta \left(t\right)+\frac{j}{\pi t}\right)\times u_{k}\left(t\right)\right]e^{-j\omega_{k}t}\right\|}_{2}^{2}+\\ {\left\|f\left(t\right)-\sum_{k=1}^{K}u_{k}\left(t\right)\right\|}_{2}^{2}+\langle \lambda \left(t\right),f\left(t\right)-\sum_{k=1}^{K}u_{k}\left(t\right)\rangle \end{array}$$

where α is the penalty factor that is used to ensure the signal reconstruction accuracy in the presence of Gaussian noise, λ is the Lagrange multiplier, and < > denotes the inner product of the vectors.

The ADMM is used to solve the above variational problem, and then the saddle point of the above augmented Lagrange function is obtained by alternately updating $u_{k}^{n+1}$, $\omega_{k}^{n+1}$, and $\lambda_{}^{n+1}$, which is the optimal solution of Equation (2). In the expression, $u_{k}^{n+1}$ is the modal function at the *n +* 1 cycle, $\omega_{k}^{n+1}$ is the center frequency of the power spectrum of the current modal function, and $\lambda_{}^{n+1}$ is the multiplication operator at the *n +* 1 cycle.

Then, the modal components $u_{k}$ and central frequencies $\omega_{k}$ and $\lambda_{k}^{n+1}$ obtained are:

(3) $${\hat{u}}_{k}^{n+1}\left(\omega \right)=\frac{\hat{f}\left(\omega \right)-\sum_{i\neq k}{\hat{u}}_{i}\left(\omega \right)+\frac{\hat{\lambda }\left(\omega \right)}{2}}{1+2\alpha {\left(\omega -\omega_{k}\right)}^{2}}$$

(4) $$\omega_{k}^{n+1}=\frac{\int_{0}^{\infty }\omega {\left|{\hat{u}}_{k}\left(\omega \right)\right|}^{2}d\omega }{\int_{0}^{\infty }{\left|{\hat{u}}_{k}\left(\omega \right)\right|}^{2}d\omega }$$

(5) $${\hat{\lambda }}^{n+1}\left(\omega \right)={\hat{\lambda }}^{n}\left(\omega \right)+\tau \left(\hat{f}\left(\omega \right)-\sum_{k=1}^{K}u_{k}^{n+1}\left(\omega \right)\right)$$

where ${\hat{u}}_{k}^{n+1}$, $\hat{f}$, and ${\hat{\lambda }}_{}^{n+1}$, respectively, represent the Fourier transform corresponding to $u_{k}^{n+1}$, f, and $\lambda_{}^{n+1}$, and τ is the noise tolerance parameter. Setting τ = 0 can achieve better noise reduction.

During the solution of alternate update, the width and frequency center of each IMF mode function are constantly updated, and the normal determination accuracy e>0 is set until the iteration stop condition shown in Equation (5) is met, and the cycle ends.

(6) $$\sum_{k=1}^{k}\left({\left\|{\hat{u}}_{k}^{n+1}-{\hat{u}}_{k}^{n}\right\|}_{2}^{2}/{\left\|{\hat{u}}_{k}^{n}\right\|}_{2}^{2}\right)<e$$

### 2.3. Wavelet Threshold Denoising (WTD)

WTD is a new denoising algorithm developed based on wavelet transform, which is suitable for the multiscale refinement of decomposed signals [4]. The selection of threshold function and the optimal threshold is the key to noise reduction. The adaptive threshold is shown in Equation (7):

(7) $$Tr_{j}=\sigma \sqrt{2\ln\left(n_{j}\right)}\;\;j=1,\ldots J$$

where $Tr_{j}$ is the *j*-level threshold, σ is the standard deviation of noise signal, and $n_{j}$ is the number of *j*-level wavelet coefficients.

There are two common threshold functions: hard threshold and soft threshold. The hard threshold method can not only keep the signal characteristics but also suppresses white noise. The mathematical expression of the hard threshold function is shown in Equation (8):

(8) $${\overset{⌢}{w}}_{s}\left(j,k\right)=\left\{\begin{array}{l} w_{s}\left(j,k\right),\left|w_{s}\left(j,k\right)\right|\geq \lambda \\ 0,\left|w_{s}\left(j,k\right)\right|\leq \lambda \end{array}\right.$$

where ${\overset{⌢}{w}}_{s}\left(j,k\right)$ is the wavelet estimation coefficient, $w_{s}\left(j,k\right)$ is the wavelet decomposition coefficient, and λ is the threshold.

### 2.4. Least Mean Square Adaptive Filtering

**Figure 2:**
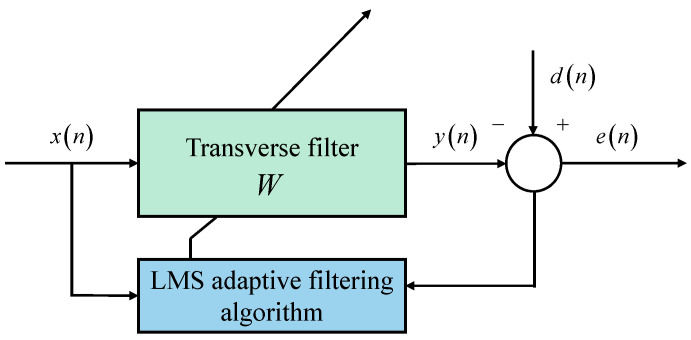
The basic structure of LMS adaptive filter.

The frequency domain filtering method is usually used to perform noise reduction processing on the signal. This method often requires the signal to have stable characteristics to suppress out-of-band noise but cannot process in-band noise. For in-band noise or non-stationary signals, the adaptive filter has a better processing effect, so the adaptive filter is an important part of the current digital signal processing applications. Among them, the least mean square (LMS) algorithm developed based on the least mean square algorithm theory is the most widely used one in adaptive filtering. The basic structure of the LMS adaptive filter is shown in Figure 2.

In Figure 2, $x\left(n\right)$ is the input signal, $y\left(n\right)$ is the output signal, $d\left(n\right)$ is the reference signal, $e\left(n\right)$ is the error signal, and W is the weight coefficient vector of the filter.

For an N-order filter, the weight coefficient vector is:

(9) $$W=\left\{w_{1},w_{2},\cdots w_{N}\right\}$$

The input vector of the filter at a certain moment is:

(10) $$X\left(n\right)=\left\{x\left(n-1\right),x\left(n-2\right),\cdots ,x\left(n-N\right)\right\}$$

Then, the error signal $e\left(n\right)$ can be expressed as:

(11) $$\begin{array}{c} e\left(n\right)=d\left(n\right)-y\left(n\right)\\ \;\;=d\left(n\right)-\sum_{i=1}^{N}w_{i}x\left(n-i\right)\\ =d\left(n\right)-X^{T}\left(n\right)W \end{array}$$

According to the goal of the filter, the purpose of the LMS algorithm is: when the filtering process is completed, the weight coefficient vector of the filter is closer to the real weight, so that the mean square error (MSE) can be minimized. Therefore, the mathematical expectation value of the squared error is selected as the objective function, namely:

(12) $$\begin{array}{c} J\left(n\right)=E\left[d^{2}\left(n\right)\right]-2E\left[d\left(n\right)X^{T}\left(n\right)\right]W\\ +W^{T}E\left[X\left(n\right)X^{T}\left(n\right)\right]W \end{array}$$

Let $P_{dx}=E\left[d\left(n\right)X^{T}\left(n\right)\right]$, $R_{xx}=E\left[X\left(n\right)X^{T}\left(n\right)\right]$, then

(13) $$J\left(n\right)=E\left[d^{2}\left(n\right)\right]-2P_{dx}W+W^{T}R_{xx}W$$

To facilitate the calculation, take the instantaneous estimated value of the objective function $J\left(n\right)$ as $\bar{J}\left(n\right)=0.5e^{2}\left(n\right)$ and derive the iterative formula of the weight coefficient vector of the filter according to the formula as:

(14) $$\begin{array}{c} W\left(n+1\right)=W\left(n\right)+\mu \left(-\frac{\partial \bar{J}\left(n\right)}{\partial W}\right)\\ =W\left(n\right)+\mu e\left(n\right)X\left(n\right) \end{array}$$

Among them, μ is the convergence factor of the algorithm, also known as the step factor, which is used to control the stability error and convergence speed of the algorithm during the filtering process.

## 3. Least Mean Square Adaptive Filter Interpolation Denoise Method Based on VMD

In the process of detection, due to factors such as temperature, propagation medium, random noise, etc., it is hard to obtain accurate ultrasonic signal characteristics from the original signal. Through the signal processing, the ultrasonic signal may be effectively extracted, and then the thickness of the material can be calculated according to the signal characteristics. This paper proposes the least mean square adaptive filter interpolation algorithm based on VMD.

**Figure 3:**
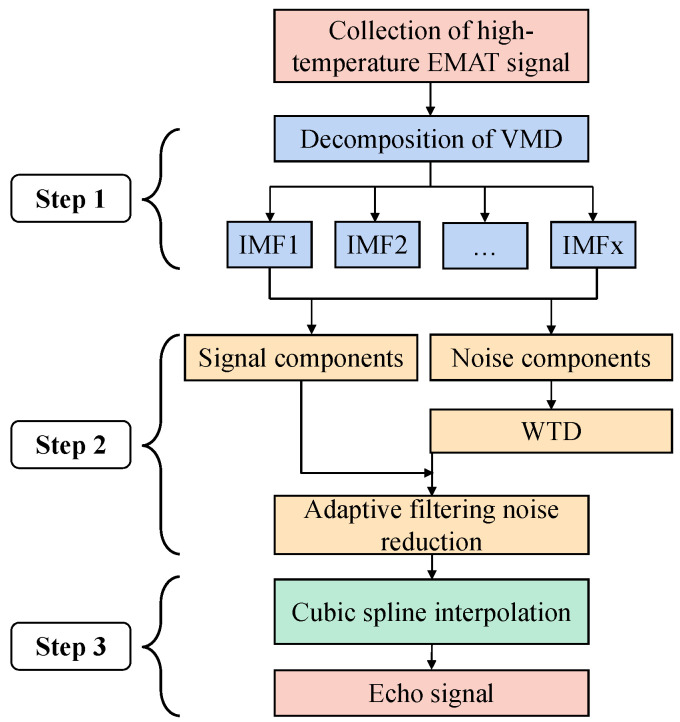
Flow chart of the proposed method.

The flow chart of the proposed method is shown in Figure 3. The algorithm can be divided into three steps:

Step 1: The original signal is decomposed by VMD to filter out the high-frequency noise and low-frequency noise.

The input signal is decomposed by VMD to obtain the IMF. The center frequencies of the IMF components are calculated by Fourier transform and arranged in order from low frequency to high frequency. Select an appropriate IMF function according to the excitation center frequency to filter out low-frequency and high-frequency noise.

Step 2: Through the adaptive filtering process, the noise components and signal components are connected to achieve noise reduction.

In the IMF obtained after VMD decomposition of the original signal, the IMF function mainly containing the excitation frequency is selected as the signal component, and the IMF function partially containing the excitation frequency is selected as the noise component. The noise component contains part of the spectrum information of useful signals, so it cannot be completely discarded. Firstly, the noise component is processed by the wavelet threshold denoising, and then it is used as the reference signal of the adaptive filtering part to perform adaptive filtering on the signal component to achieve the noise reduction effect.

Step 3: Extract the echo signal and obtain the echo information.

After the ultrasonic signal with high SNR is obtained, the time interval between waveforms can be obtained by extracting the envelope, but this method cannot achieve high measurement accuracy when processing the actual signal. As an improved piecewise interpolation, cubic spline interpolation not only has the advantages of a simple algorithm but also makes up for the disadvantage of poor smoothness of piecewise interpolation. In addition, the cubic spline interpolation method can be used to obtain more time characteristics of signals. This paper uses this advantage to apply the method to noise reduction signal, further refining the sampling points based on each original sampling point, so that the filtering curve is smoother and the effect is better [28].

## 4. Experimental Setup

### 4.1. High-Temperature Experimental Setup

**Figure 4:**
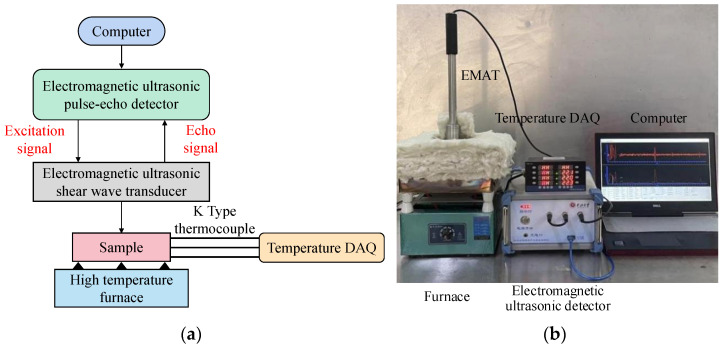
High-temperature experimental system. (**a**) Schematic diagram; (**b**) experiment setup.

The high-temperature experimental setup used in this study is given in Figure 4, which consists of a computer (Dell, Dell Computer, Round Rock, TX, USA), self-developed ultrasonic detector, self-developed EMAT, furnace (2500 W, Electric Company, Hongkong, China), and temperature DAQ (KCM-XJ8, Instrument and instrument factory, Beijing, China) with the K-type thermocouple (K-type, Instrument and instrument factory, Beijing, China). The computer sends detection parameters such as excitation voltage, number of cycles, and center frequency to the pulsed electromagnetic ultrasonic detector through the USB interface. The pulsed electromagnetic ultrasonic detector controls the transmitting circuit to output an excitation signal with a transient peak power and frequency from 0.5 to 15 kW and 1 to 4.5 MHz according to the detection parameters. The detection signal is received by the transducer, amplified by the electromagnetic ultrasonic detector, and stored in the computer in the form of a digital signal.

The designed coil adopts a spiral coil structure, with an inner diameter of 2.25 mm and an outer diameter of 18.25 mm. It consists of 25 coils with a coil spacing of 0.12 mm. In order to ensure electromagnetic shielding, the magnet and the coil are separated by 2 mm thick copper foil. Lifting the coil by 2 mm and protecting it in a ceramic sleeve can eliminate the influence of the heated specimen on the coil. To overcome the high-temperature effect, the EMAT used in this study are modified to fit the hot environment. The BNC joint, EMAT house, and magnet are made by high-temperature-resistant materials. Additionally, double built-in thermal insulation layers are inserted to protect the copper foil in the EMAT, which effectively reduces the influence of the heat source on the internal coil and permanent magnet. After testing, the maximum temperature the high-temperature EMAT can work at is 800 °C.

The sample materials used in this study are Cr25Mo3Ti and 12CrMo, these two materials are widely used in industrial construction and other fields. The sample was laid on the furnace for temperature controlling, which changes from 25 to 700 °C. The EMAT was put on the top surface of the sample to excite and receive ultrasonic waves. To eliminate the heat transfer effect in the air on the testing results, 5 cm-thick thermal insulation material was used to surround the sample. Three K-type thermocouples insert into the workpiece to monitor the sample temperature. The first one is attached on the top surface of the sample. The second one lies in the middle of the side surface. The third one inserts in the contact surface between the sample and furnace. To ensure the thermal stability of the sample, the temperature difference of the three K-type thermocouples should be less than 10 °C.

**Figure 5:**
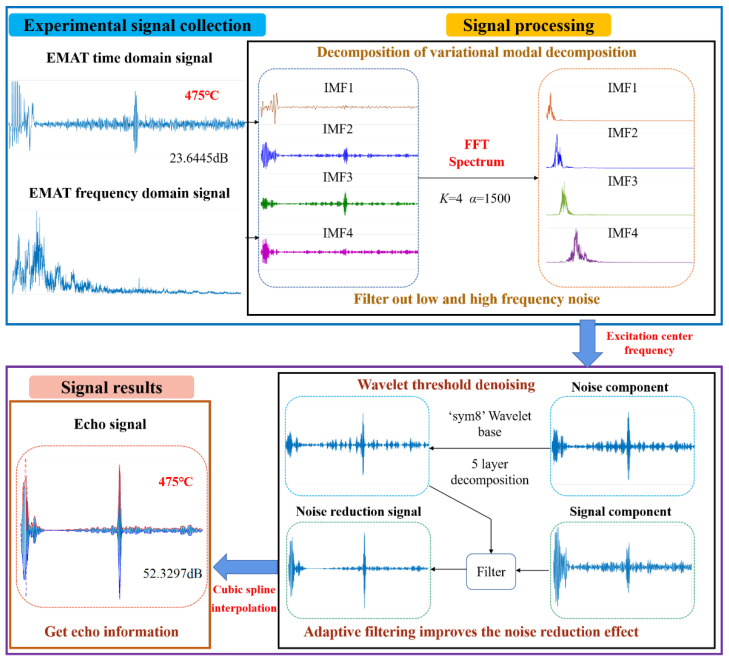
Denoising process diagram of least mean square adaptive filtering interpolation based on VMD.

In the ultrasonic detection signal, there are electronic noises from the circuit boards of the instrument and structure noise formed by the scattering of ultrasonic waves in the high-temperature specimen. Due to the relatively low efficiency of the current electromagnetic ultrasonic transducer, the strength of the detection signal is weak, and the receiving circuit is susceptible to interference. This results in more noise and clutter in the detected data. Pulsating noise exists widely in ultrasonic signals, and the pulsating noise is mainly concentrated in the low-frequency domain. The principle of electromagnetic ultrasonic testing is the pulse–echo method. The function of the ultrasonic probe is to transmit and receive echoes. Therefore, the electromagnetic ultrasonic transducer will inevitably have a reverberation phenomenon, and this noise will cause random fluctuations in the signal. This leads to a deviation in the extraction of extreme points in the process of extracting the envelope, so the noise should be filtered out. It can be seen from the spectrum diagram after VMD decomposition in Figure 5 that there are some high-frequency electrical noises in the echo signal. When the transmitting signal frequency is known, the effective signal frequency of the echo signal is distributed around the central frequency of the transmitting signal. The excessively high frequency part is obviously invalid high-frequency white noise, which leads to poor SNR of the echo signal and therefore needs to be filtered. The distribution of the two kinds of noise is non-overlapping with the frequency of the effective signal. According to this characteristic, this paper decomposes the EMAT signal by VMD.

### 4.2. Thickness Measurement Principle of EMAT

Ultrasonic pulses emitted by the EMAT pass through the test sample and are reflected to the EMAT on the material interface. At different temperatures, the propagation velocity of ultrasonic waves in a given medium can be obtained by calibrating or consulting the material sound velocity library. Therefore, by measuring the propagation time of ultrasonic waves in the material, the thickness of the material can be calculated using Equation (15).

(15) $$h=v\left(T\right)\times \frac{t_{2}-t_{1}}{2}$$

where $v\left(T\right)$ is the shear wave sound velocity of the materials at temperatures *T*, *h* is the thickness of the material, and *t*_1_ and *t*_2_ are the time of the two adjacent echoes.

### 4.3. Parameters Decision

In the process of data processing, the choice of parameters is very important. In the VMD decomposition, the secondary penalty term α is set to a median value of 1500 to obtain better convergence [11]. Due to the strong noise, the Lagrange multiplier is set to a median value of 0. The convergence accuracy ε is 1 × 10^−6^. The traditional VMD algorithm needs to set the value of K in advance, which means that the signal will be decomposed into K modes. If K is too small, modal component information will be lost, resulting in frequency aliasing. For a *K* with too large a value, it will cause over-decomposition. After many repeated experiments, it is found that when K = 4, the decomposition effect of the signal is the best.

In WTD, according to the wavelet basis function and the characteristics of the signal to be measured, the SymN wavelet is selected, and the maximum value N = 8 is selected to directly ensure its optimal characteristics. In addition, the noise level estimates at each layer of the wavelet decomposition are adjusted using the sqtwlog rule.

### 4.4. Signal-to-Noise Ratio Calculation

To compare noise reduction performance of different signal processing methods, the peak SNR is introduced [29]. The mathematical expression of PSNR is as follows:

(16) $$SNR_{dB}=20\mathrm{lg}\frac{A_{signal}}{A_{noise}}$$

where $SNR_{dB}$ is the SNR of the signal, $A_{signal}$ is the maximum amplitude value in an intercepted wave packet, and $A_{noise}$ is the average value of several noise amplitudes in a region after the echo.

## 5. Results Analysis and Discussion

### 5.1. Signal Processing

In this study, a set of detection signals of the material 12CrMo at 475 °C are taken as an example, and the specific process of noise reduction is described in detail. The pulsed electromagnetic ultrasonic detector is controlled by the host computer software to excite two cycles. The excitation voltage is 250 V, and the excitation signal with the center frequency of 3.25 MHz realizes electro-acoustic conversion on the surface of the tested sample and generates ultrasonic waves. The noise reduction method proposed in this paper is applicable to any condition, rather than a fixed excitation frequency. Different excitation frequencies result in different center frequencies of ultrasonic echo signals. Figure 5 shows the time and frequency domain diagram of the original signal from the tested sample.

Due to the excitation frequency of 3.25 MHz that was used, both IMF2 and IMF3 decomposed by VMD contain useful signals. Most of the useful signals are included in IMF2. Select IMF2 where the useful signal is located as the signal component of adaptive filtering and IMF3 as the noise component, as shown in Figure 5. The echo signal in this paper is a non-stationary signal. Consider the advantages of wavelet noise reduction, such as low entropy, variable resolution, irrelevance, wavelet base selection diversity, and processing non-stationary signals, removing noise similar to the signal frequency. Due to the advantages of other aspects, this paper adopts the wavelet threshold noise reduction. Since the phase distortion affects the echo time accuracy, this paper selects the SymN wavelet, directly taking the maximum value of N = 8 to ensure its optimal characteristics. The decomposition level is set to 5. These choices are based on implementation in many replicated experiments. In addition, using a hard threshold function, the “sqtwolog” rule adjusts the noise level estimates for each layer of the wavelet decomposition.

The signal is further processed by adaptive filtering, and the signal whose noise component is denoised by the WTD is used as a reference signal to denoise the signal component so that the obtained reference signal can better reflect the noise information in the signal. The obtained reduction noise signal also preserves the local features of the ultrasound signal, as shown in Figure 5. On this basis, the adaptively filtered signal is processed by cubic spline interpolation, and the echo signal of the final signal is extracted, as shown in Figure 5.

### 5.2. Validation of Proposed Method through Thickness Measurement

**Figure 6:**
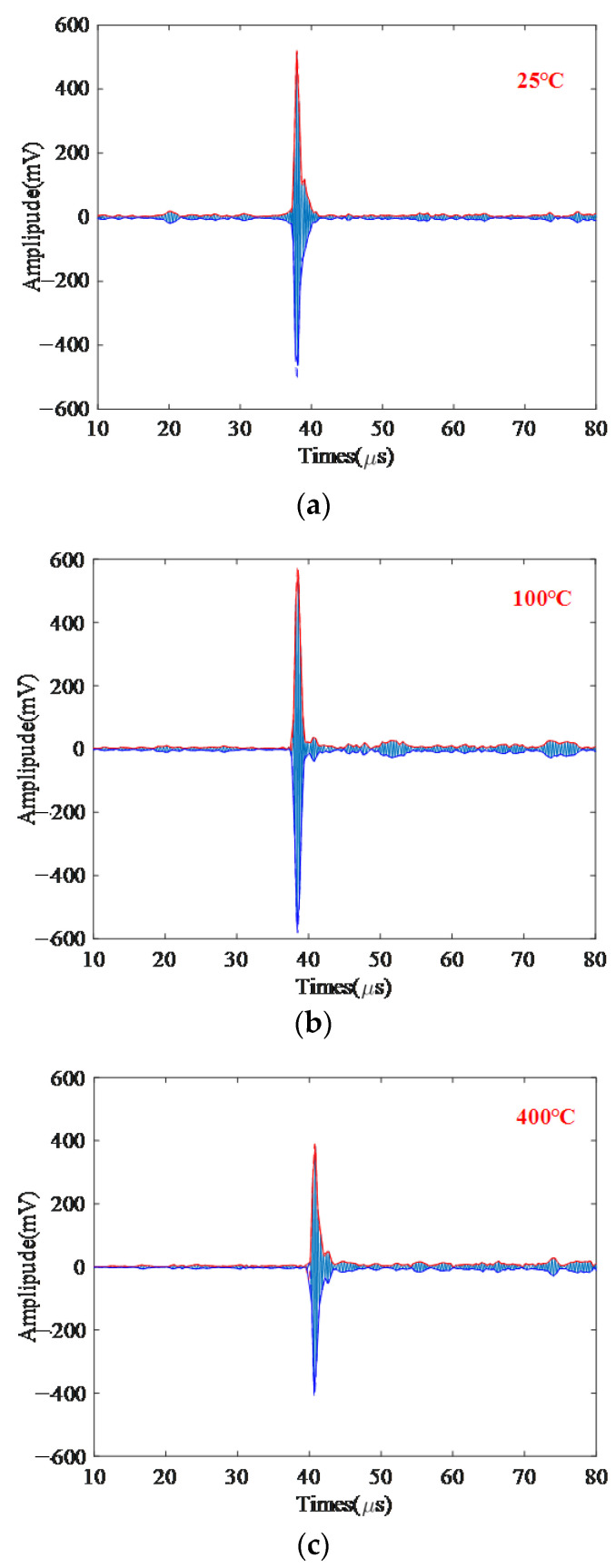

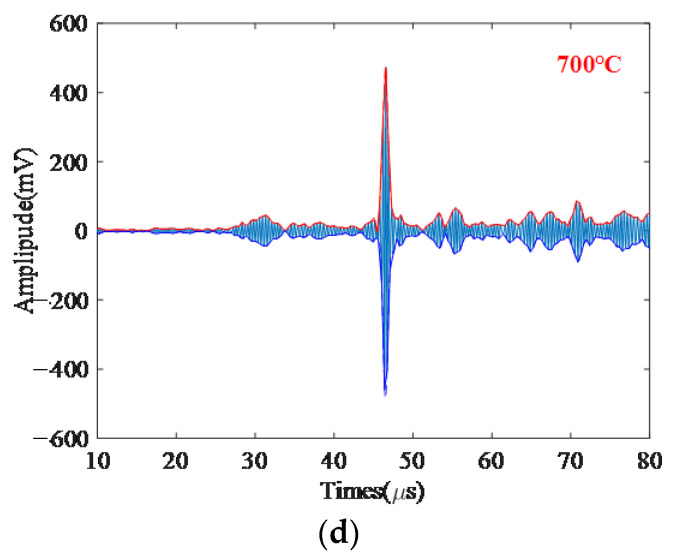
Signals of Cr25Mo3Ti after cubic spline interpolation at different temperatures (**a**) 25, (**b**) 100, (**c**) 400, and (**d**) 700 °C.

In this paper, the accuracy of thickness measurement is used as a new index to verify the performance of the algorithm. Cr25Mo3Ti, with a thickness of 60 mm, was placed in a heating furnace and heated from 25 to 100, 200, 300, 400, 500, 600, and 700 °C, respectively. Adaptive filtering noise reduction based on VMD is performed on the high-temperature original signals at different temperatures. Then, the echo signals are extracted. The echo signals at different temperatures are subjected to cubic spline interpolation processing to extract time information. The waveform after cubic spline interpolation is shown in Figure 6.

It is known that the actual thickness of the material Cr25Mo3Ti at 25 °C is 60 mm. The electromagnetic ultrasonic signal of the material Cr25Mo3Ti from 25 °C to 700 °C is processed by the method proposed in this paper. By measuring the propagation time of ultrasonic waves in the material, the thickness of the material Cr25Mo3Ti at different temperatures was calculated, as shown in Table 1. It can be seen from Table 1 that the thickness measurement accuracy of the method proposed in this paper is kept within 2%, and the online thickness measurement at high temperature is realized.

### 5.3. Comparison with Other Noise Reduction Methods

To compare the noise reduction effect of the adaptive filtering based on VMD proposed in this paper, this paper selects the same group of signals with an original signal of 23.6445 dB at 475 °C of material 12CrMo for noise reduction effect assessment.

**Figure 7:**
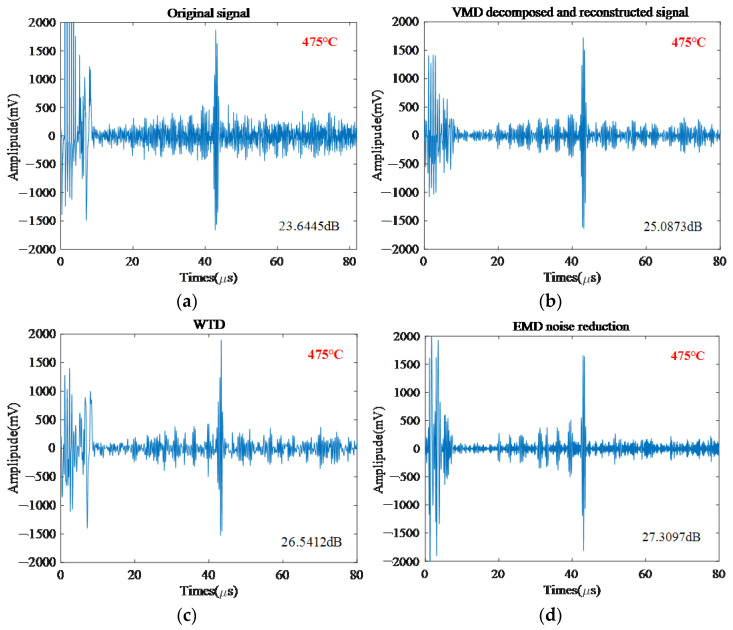
12CrMo (**a**) original signal, (**b**) VMD decomposed and reconstructed signal, (**c**) WTD, and (**d**) EMD noise reduction at 475 °C.

Figure 7, respectively, shows the original electromagnetic ultrasonic signal and the denoising signal processed by the VMD, WTD, and EMD methods. As can be seen in Figure 7, the denoising method proposed in this paper is superior to the other three methods, it cannot only preserve the useful part of the signal but also effectively remove most of the noise in the target signal. This is because the VMD method is more sensitive to noise. When there is noise, modal aliasing may occur in the decomposition, and the ability to suppress noise is weak. When the WTD method is used alone, it still cannot achieve effective noise reduction. WTD combined with the VMD method is better than using it alone. However, the EMD method itself has the shortcomings of modal aliasing and end-point effect. Compared with VMD, EMD was more sensitive to noise. The filtering effect is prone to waveform distortion, and the original characteristics of the signal cannot be preserved to the greatest extent. However, the method in this paper is different from the previous traditional independent noise reduction idea. Through the adaptive filtering process, the dominant component of the noise and the dominant component of the signal are linked, the components of the useful signal will not be lost, and the ability to suppress noise is strong. Therefore, the proposed method can greatly improve the SNR of the high-temperature EMAT signal, and at the same time perform cubic spline interpolation processing on the noise-reduced signal, which can further improve the echo extraction accuracy of the body wave, which is beneficial to the online thickness measurement at high temperature.

From Figure 7, the noise reduction capability of each method can be seen intuitively. For further quantitative comparison, combined with the actual situation, different noise reduction methods are used to denoise the EMAT signals of the same group. At the same time, the SNR is introduced as the basis for judging the denoising ability, as shown in Table 2. It can be seen from Table 2 that the denoising effect of the method proposed in this paper is obviously better than the other three methods, and the SNR of the denoised signal is greatly improved. The denoising effects of the WTD method and EMD method are similar, and the improvement of the SNR is small, but the noise reduction effect is slightly better than that of the VMD method. The results show that the proposed method improves the SNR of high-temperature EMAT signals by two times and is more suitable for processing high-temperature EMAT signals.

### 5.4. High-Temperature Robustness of the Proposed Method

**Figure 8:**
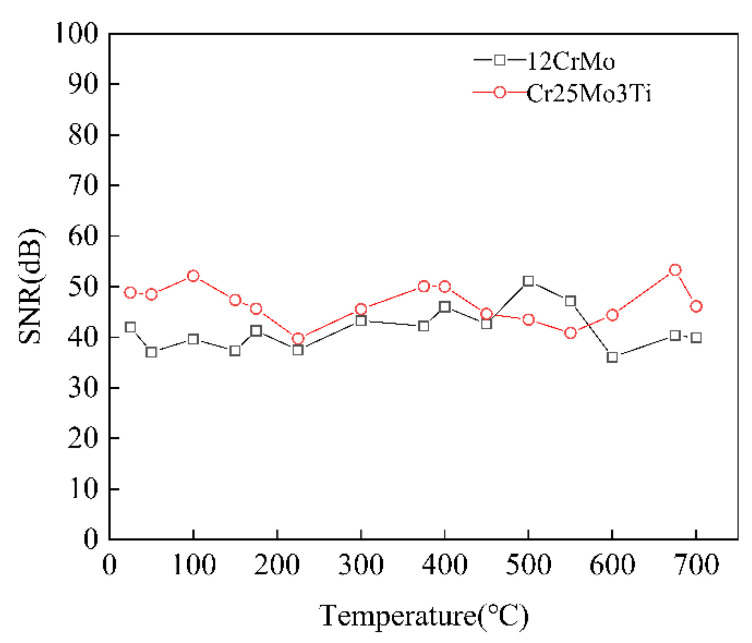
SNR of Cr25Mo3Ti and 12CrMo signals at different temperatures after AFIV processing.

The EMAT signals of Cr25Mo3Ti and 12CrMo from 25 to 700 °C were denoised by the AFIV denoising method. The SNR result of the denoising signal is shown in Figure 8. It can be seen that the denoising effect of AFIV method is not affected by temperature and basically keeps within a stable range. The effectiveness of AFIV noise reduction method is verified.

**Figure 9:**
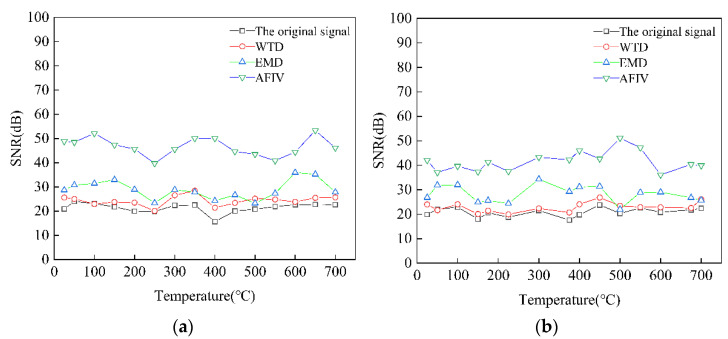
SNR of (**a**) Cr25Mo3Ti and (**b**) 12CrMo signals at different temperatures after different noise reduction method processing.

To verify the applicability of the denoising method proposed in this paper, ultrasonic detection was carried out on Cr25Mo3Ti and 12CrMo materials in the range of 25 to 700 °C. During the experiment, it must be noted that all samples were heated from the furnace bottom, and temperature measurements were made on the surface of each sample. As shown in Figure 9, the electromagnetic ultrasonic signals measured at intervals of 50 °C were selected and processed by different denoising methods, which verified that the denoising method proposed in this paper is suitable for the selected temperature range, and the SNR greatly improved.

As can be seen from Figure 9, compared with the other three noise reduction methods, the SNR of the EMAT signal obtained by the noise reduction method proposed in this paper is 40~60 dB in the range of 25 to 700 °C for the materials Cr25Mo3Ti and 12CrMo. The SNR of the signal is greatly improved. The SNR of original signal is the lowest, maintained at about 20 dB. Compared with the original signal, the SNR of the wavelet threshold noise reduction method and the EMD method are improved to a certain range. The experimental results show that the noise reduction method has a good noise reduction effect on the EMAT signals of materials Cr25Mo3Ti and 12CrMo, and the echo signal is more obvious, which is more conducive to extracting the echo signal information.

## 6. Conclusions

This paper proposes the least mean square adaptive filtering interpolation denoising method based on VMD (AFIV). First, decompose the original signal by VMD, filter out low- and high-frequency noise, and use wavelet threshold denoising to process the noise component after decomposing the signal by VMD. Then, it is used as the reference signal of the adaptive filtering part to complete the noise reduction in the signal component to realize the final adaptive filtering process. Finally, cubic spline interpolation is performed on the noise reduction signal after adaptive filtering, and the echo signal is extracted. This method breaks through the traditional idea of the independent processing of signal components and uses adaptive filtering technology to connect useful information between noise components and signal components. Compared with other methods, this method can adaptively adjust the center frequency of each mode, improve the SNR of high-temperature EMAT signals, and further improve the echo extraction accuracy of body waves. By analyzing the experimental results of different materials at different temperatures, it is shown that this method has a good noise reduction effect on the EMAT signal with low SNR in the range of 25 to 700 °C, making the echo information more obvious, and can achieve online thickness measurement at high temperature with an error of less than 2%.

## Figures and Tables

**Table 1:** Thickness measurement of Cr25Mo3Ti at different temperatures.

Temperature (°C)	Acoustic Speed [30] (m/s)	Calculated Thickness (mm)	Actual Thickness (mm)	Error (%)
25	3246	61.122	60	1.87
100	3122	60.223	60.975	1.23
200	3093	59.911	60.987	1.76
300	3038	60.213	60.994	1.28
400	2940	59.917	61.001	1.77
500	2870	60.040	61.013	1.59
600	2795	62.115	61.033	1.77
700	2656	61.805	61.062	1.21

**Table 2:** Comparison of original signal noise reduction performance of various methods at 475 °C.

Denoising Method	VMD	WTD	EMD	AFIV
SNR/dB	25.0873	26.5412	27.3097	52.3297

## Data Availability

Data sharing not applicable.

## References

[1] Brizuela J., Camacho J., Cosarinsky G., Iriarte J., Cruza J. (2019). Improving elevation resolution in phased-array inspections for NDT. NDT E Int..

[2] Song S., Ni Y. (2018). Ultrasound imaging of pipeline crack based on composite transducer array. Chin. J. Mech. Eng..

[3] Baba A., Searfass C., Tittmann B. (2010). High temperature ultrasonic transducer up to 1000 °C using lithium niobate single crystal. Appl. Phys. Lett..

[4] Donoho D., Johnstone I. (1995). Adapting to unknown smoothness via wavelet shrinkage. J. Am. Stat. Assoc..

[5] Legendre S., Goyette J., Massicotte D. (2001). Ultrasonic NDE of composite material structures using wavelet coefficients. NDT E Int..

[6] Huang N., Shen Z., Long S., Wu M., Shih H., Zheng Q., Yen N., Tung C., Liu H. (1998). The empirical mode decomposition and the Hilbert spectrum for nonlinear and non-stationary time series analysis. Proc. Math. Phys. Eng. Sci..

[7] Sun M., Shen Y., Zhang W. A wavelet threshold denoising method for ultrasonic signal based on EMD and correlation coefficient analysis. Proceedings of the 2010 3rd International Congress on Image and Signal Processing.

[8] Xu H., Xu C., Zhou S. A new ultrasonic guided wave signal processing method for UNDE of laminated composite material. Proceedings of the 2010 International Conference on Mechanic Automation and Control Engineering.

[9] Wu Z., Huang N. (2009). Ensemble empirical mode decomposition: A noise-assisted data analysis method. Adv. Adapt. Data Anal..

[10] Zhang J., Qin X., Yuan J., Wang X., Zeng Y. (2021). The extraction method of laser ultrasonic defect signal based on EEMD. Opt. Commun..

[11] Dragomiretskiy K., Zosso D. (2014). Variational mode decomposition. IEEE Trans. Signal Process..

[12] Si D., Gao B., Guo W., Yan Y., Tian G., Yin Y. (2019). Variational mode decomposition linked wavelet method for EMAT denoise with large lift-off effect. NDT E Int..

[13] Abdessalem B., Farid C. (2020). Resolution Improvement of Ultrasonic Signals Using Sparse Deconvolution and Variational Mode Decomposition Algorithms. Russ. J. Nondestruct. Test..

[14] Li F., Zhang B., Verma S., Marfurt K. (2018). Seismic signal denoising using thresholded variational mode decomposition. Explor. Geophys..

[15] Hu H., Zhang L., Yan H., Bai Y., Wang P. (2019). Denoising and base-line drift removal method of MEMS hydrophone signal based on VMD and wavelet threshold processing. IEEE Access.

[16] Ram R., Mohanty M.N. (2018). Performance analysis of adaptive variational mode decomposition approach for speech enhancement. Int. J. Speech Technol..

[17] Gu X., Chen C. (2017). Rolling bearing fault signal extraction based on stochastic resonance-based denoising and VMD. Int. J. Rotating Mach..

[18] Kogia M., Gan T., Balachandran W., Livadas M., Kappatos V., Szabo I., Mohimi A., Round A. (2016). High temperature shear horizontal electromagnetic acoustic transducer for guided wave inspection. Sensors.

[19] Shi W., Chen W., Lu C., Cheng J., Chen Y. (2021). Application of chirp pulse compression technique to a high-temperature EMAT with a large lift-off. Int. J. Appl. Electromagn. Mech..

[20] Basili M., Casini P., Morelli L., Vestroni F. (2021). Vibration Mitigation of Rail Noise Barriers by Hysteretic Absorbers. J. Appl. Comput. Mech..

[21] Tsakonas E.E., Sidiropoulos N.D., Swami A. (2008). Optimal particle filters for tracking a time-varying harmonic or chirp signal. IEEE Trans. Signal Process..

[22] Lei Z., Su W., Hu Q. (2019). Multimode Decomposition and Wavelet Threshold Denoising of Mold Level Based on Mutual Information Entropy. Entropy.

[23] Wei X., Feng G., Qi T., Guo J., Li Z., Zhao D., Li Z. (2022). Reduce the Noise of Transient Electromagnetic Signal Based on the Method of SMA-VMD-WTD. IEEE Sens. J..

[24] Ribichini R., Cegla F., Nagy P.B., Cawley P. (2010). Modelling of electromagnetic acoustic transducers operating on ferromagnetic materials. Am. Inst. Phys..

[25] Hirao M., Ogi H. (2003). EMATs for Science and Industry: Non Contacting Ultrasonic Measurements.

[26] Wang S., Li Z., Li P., Liu X., Zhai G. (2014). Numerical and experimental evaluation of the receiving performance of meander-line coil EMATs. Nondestruct. Test. Commun..

[27] Ribichini R., Nagy P.B., Ogi H. (2012). The impact of magnetostriction on the transduction of normal bias field EMATs. NDT E Int..

[28] Li S., Wang H., Guo R., Zhao J., Zheng K., Xu J., Chen S. (2018). Nondestructive testing thickness measurement by laser ultrasound under high temperature. Int. J. Light Electron. Opt..

[29] Lunn N., Dixon S., Potter M.D.G. (2017). High temperature EMAT design for scanning or fixed point operation on magnetite coated steel. NDT E Int..

[30] Zheng Y., Li Z., Zhou J., Zhang Z. (2021). Study on the Change Law of Transverse Ultrasonic Velocity in a High Temperature Material. Res. Nondestruct. Eval..

